# An engineered bispecific DNA-encoded IgG antibody protects against *Pseudomonas aeruginosa* in a pneumonia challenge model

**DOI:** 10.1038/s41467-017-00576-7

**Published:** 2017-09-21

**Authors:** Ami Patel, Antonio DiGiandomenico, Ashley E. Keller, Trevor R. F. Smith, Daniel H. Park, Stephanie Ramos, Katherine Schultheis, Sarah T. C. Elliott, Janess Mendoza, Kate E. Broderick, Megan C. Wise, Jian Yan, Jingjing Jiang, Seleeke Flingai, Amir S. Khan, Kar Muthumani, Laurent Humeau, Lily I. Cheng, Leslie Wachter-Rosati, C. Kendall Stover, Niranjan Y. Sardesai, David B. Weiner

**Affiliations:** 1 The Wistar Institute of Anatomy & Biology, Philadelphia, PA 19104 USA; 2 MedImmune, Gaithersburg, MD 20878 USA; 3 Inovio Pharmaceuticals, Plymouth Meeting, PA 19462 USA

## Abstract

The impact of broad-spectrum antibiotics on antimicrobial resistance and disruption of the beneficial microbiome compels the urgent investigation of bacteria-specific approaches such as antibody-based strategies. Among these, DNA-delivered monoclonal antibodies (DMAbs), produced by muscle cells in vivo, potentially allow the prevention or treatment of bacterial infections circumventing some of the hurdles of protein IgG delivery. Here, we optimize DNA-delivered monoclonal antibodies consisting of two potent human IgG clones, including a non-natural bispecific IgG1 candidate, targeting *Pseudomonas aeruginosa*. The DNA-delivered monoclonal antibodies exhibit indistinguishable potency compared to bioprocessed IgG and protect against lethal pneumonia in mice. The DNA-delivered monoclonal antibodies decrease bacterial colonization of organs and exhibit enhanced adjunctive activity in combination with antibiotics. These studies support DNA-delivered monoclonal antibodies delivery as a potential strategy to augment the host immune response to prevent serious bacterial infections, and represent a significant advancement toward broader practical delivery of monoclonal antibody immunotherapeutics for additional infectious pathogens.

## Abstract (web summary)

DNA-delivered monoclonal antibodies (DMAbs) can be produced by muscle cells in vivo, potentially allowing prevention or treatment of infectious diseases. Here, the authors show that two DMAbs targeting *Pseudomonas aeruginosa* proteins confer protection against lethal pneumonia in mice.

## Introduction

The impact of growing antimicrobial resistance (AMR) arising from broad-spectrum antibiotic exposure threatens the antibiotic cornerstone of modern medicine. The global rate of mortality from AMR infections is estimated to be >700 000/year and expected to rise over the next decade^1^, and the CDC reports over 2 million AMR cases in the United States each year (>23 000 deaths/year)^2^. The lack of progress in the development of new antibiotic classes and new understanding of host-pathogen interactions is prompting interest in microbiome-friendly approaches that engage the immune system with minimal disruption of the normal microbiota. One such approach is the investigation of antibody technology for preventing and treating bacterial infections. Therapeutic monoclonal antibodies (mAbs) are now a mainstay to treat several diseases, including primary immunodeficiencies, autoimmune disorders, asthma, cancer, and graft transplantation (reviewed in refs.^3–8^). However, only two mAbs are approved for treatment of infectious diseases in humans, palivizumab (targeting respiratory syncytial virus) and bezlotoxumab (targeting *Clostridium difficile* toxin B). Raxibacumab and obiltoxaximab, both targeting inhalation anthrax, were approved under the Food and Drug Administration ﻿(FDA) animal rule because they could not be reasonably tested for efficacy in humans. Several other infectious disease mAb candidates are currently being evaluated in human clinical trials (clinicaltrials.gov, reviewed in refs ^9, 10^). Yet, there are several technological hurdles with cost-effective mAb-based delivery that impede a paradigm shift from broad-spectrum approaches to pathogen-specific, targeted prevention and treatment of bacterial infections. These limitations to protein IgG administration include dosage (in the range of mg/kg), mAb production challenges, and significant costs associated with current manufacturing technology.

DNA plasmid delivery offers a novel approach to circumvent the hurdles of traditional mAb delivery through direct, in vivo immunoglobulin (Ig) production. DNA-delivered monoclonal antibodies (DMAb) are transiently expressed and secreted by skeletal muscle cells and directly enter the systemic circulation to prevent virulence while promoting bacterial clearance. This is facilitated though specific Ig heavy and light chain nucleotide sequence optimizations, formulation development, and by recent advances in in vivo electroporation (EP) technology that has directly lead to increased DNA uptake into cells, translating to significantly enhanced DNA expression in vivo^11^.

Employing this pathogen-specific approach we utilized the DMAb platform to deliver two well-characterized mAbs targeting *Pseudomonas aeruginosa*, an environmentally ubiquitous bacterium that can cause life-threatening infections in critically ill and immunocompromised patients. In addition, intrinsic antibiotic resistance via elaborate drug efflux systems, the acquisition of antibiotic resistance through horizontal gene transfer, as well as the ability to form biofilms makes *P. aeruginosa* a formidable foe in seriously ill patients. Two promising mAb targets against *P. aeruginosa* include the Psl exopolysaccharide and the type 3 secretion (T3S) injectisome PcrV protein. Psl is a highly abundant serotype-independent polysaccharide important for host cell attachment, biofilm formation, and immune evasion^12–14^. The T3S injectisome induces intoxication by injection of multiple effectors directly into the host cell cytoplasm where they interact with a multitude of eukaryotic co-factors. PcrV is thought to sit at the apex of the molecular syringe and plays a prominent role, along with other bacterial proteins, in host cell membrane pore formation^15^. Protective mAbs to both targets were recently described, with each alone exhibiting potent activity in multiple *P. aeruginosa* infection models^16, 17^. In addition, clinical candidate MEDI3902, a bispecific mAb targeting Psl and PcrV, was recently described and shown to exhibit synergistic protective activity when compared to the individual mAbs or a parental mAb mixture^18^. DMAb delivery as an alternative strategy to bypass the need for protein IgG could be highly beneficial for administration of pathogen-specific mAbs to at-risk target populations. Furthermore, the low cost of DMAb production, the favorable safety profile of DNA delivery in humans, and the ease of administration^19^ would benefit a wider population of individuals that are at risk for infection.

Here, we describe the development and analysis of synthetic DMAbs consisting of a monospecific anti-PcrV IgG (DMAb-αPcrV) and clinical candidate bispecific antibody MEDI3902 (DMAb-BiSPA) for in vivo production and activity. We observe that DMAb production in vivo can rapidly yield functional and protective titers for both constructs. These DMAbs can persist and have similar potency to bioprocess-produced mAbs, along with comparable prevention of *P. aeruginosa* colonisation of major organs. We also demonstrate that skeletal muscle cells can support production and secretion of functional, engineered bispecific IgG. This finding highlights the flexibility of the host cellular machinery to form novel, non-natural Ig forms in vivo and expands the possibilities for encoding additional pathogenic-specific affinities. Furthermore, we show that DMAb-BiSPA exhibits enhanced protective activity with antibiotic treatment in a lethal pneumonia model. Taken together, our results suggest that DNA delivery of full length IgG mAbs is a promising strategy for prevention of serious bacterial infections and possibly for other therapeutic indications.

## Results

### Design and in vitro expression of anti-*P. aeruginosa* DMAbs

**Fig. 1:**
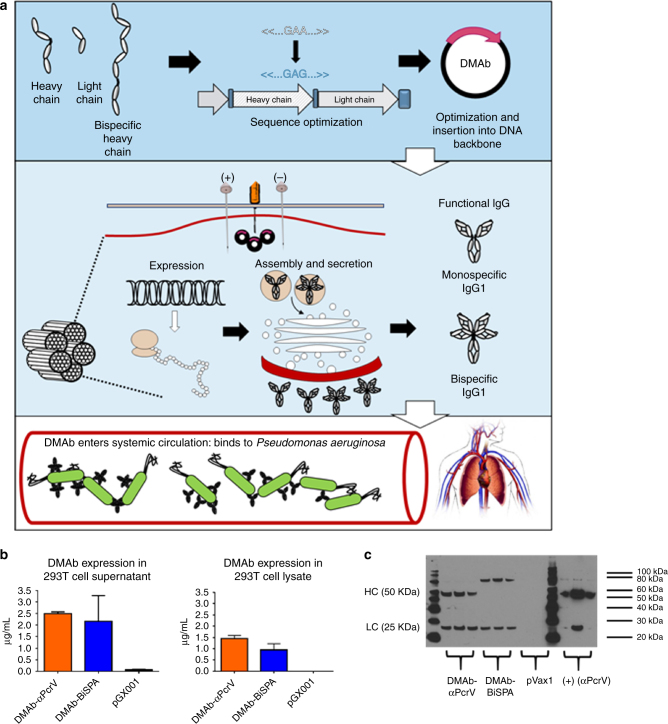
Schematic diagram of DMAb delivery and in vitro expression. **a** DMAbs were designed to encode IgG antibody heavy and light chains of monoclonal antibody clones V2L2MD and MEDI3902, resulting in the DMAb-αPcrV and DMAb-BiSPA constructs. The optimized DMAb constructs are administered to mice by in vivo IM-EP, and muscle cells being to synthesize an produce mAb. Fully functional DMAb is secreted and enters the systemic circulation. (The image of the human circulatory system is licensed under a Creative Commons Share-Alike 3.0 License (CC-BY-SA) https://creativecommons.org/licenses/by-sa/3.0/ and can be found at thecardiovascularsystem.wikispaces.com/HOME). **b** HEK 293 T cells were transfected with 1 µg/well of DMAb-αPcrV, DMAb-BiSPA, or control pGX0001. Supernatant and cell lysates were harvested after 48 h. Samples were assayed for human IgG. *Error bars* represent the standard deviation. **c** A Western blot was performed with cell lysates from transfected cells. 10 µg total cell lysate was loaded in each lane and run on an SDS-PAGE gel, followed by transfer onto a nitrocellulose membrane. The membrane was probed with a goat anti-human IgG H + L antibody, conjugated to HRP. Samples were developed using an ECL chemiluminescence kit and visualized on film

We selected two anti-*P. aeruginosa* mAb genes to be re-encoded for optimal expression into a DNA expression vector system based on their previously described potent protective in vivo activity against lethal *P. aeruginosa* infection^17, 18^. The nucleotide and amino acid sequences of the human immunoglobulin gamma 1 (IgG1) heavy and light chains (Fab and Fc portions) were optimized taking into consideration both human and mouse codon bias, and encoded as a single, polycistronic unit in the pGX0001 DNA plasmid backbone, resulting in two constructs: DMAb-αPcrV and DMAb-BiSPA (Fig. 1a). The heavy and light chains are expressed as a single mRNA transcript and then cleaved post-translationally at a porcine teschovirus-1 2A (P2A) cleavage site. A furin cleavage site (RGRKRRS) was also included to ensure complete removal of the P2A from the final in vivo produced antibody.

We first assessed the ability for each construct to express full length human IgG1 antibody following in vitro transfection of HEK293T cells. The DMAb-transfected cells and supernatants were harvested after 48 h and a total IgG enzyme-linked immunosorbent assay (ELISA) was performed on the cell lysates and in the medium, which verified DMAb IgG production and secretion (Fig. 1b). A Western blot was also performed to confirm that both antibody heavy and light chains were expressed. The heavy chain for the bispecific DMAb runs at a higher molecular weight as it encodes two variable region specificities (Fig. 1c). pGX0001 DNA vector was included as a negative control and purified anti-PcrV IgG1 as a positive control.

### Expression of DMAb-αPcrV and DMAb-BiSPA in mice

**Fig. 2:**
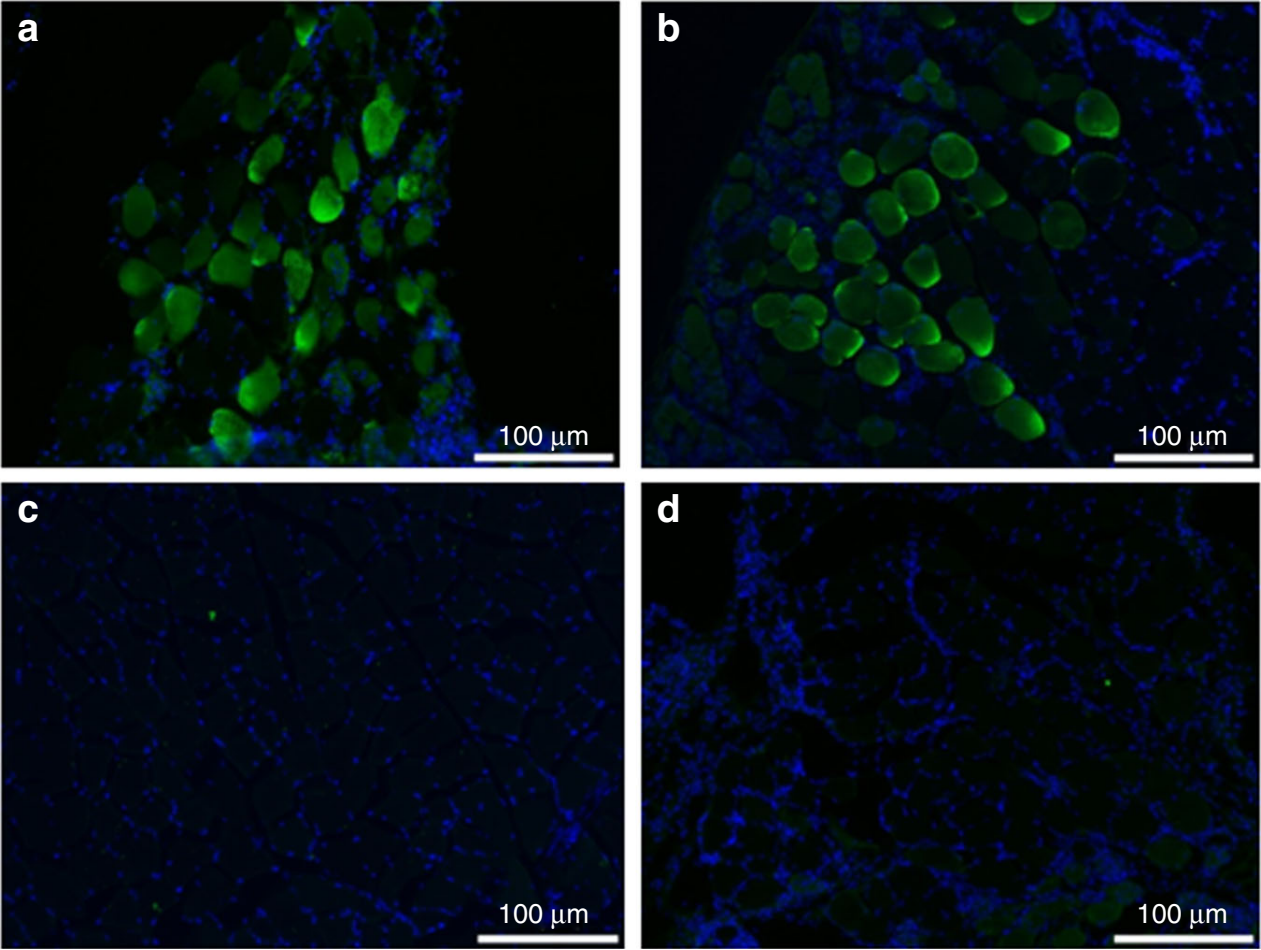
Expression of DMAb-αPcrV and DMAb-BiSPA in mouse skeletal muscle. BALB/c mice received a DNA injection, in the TA muscle with DMAb-αPcrV or DMAb-BiSpA DNA followed by in vivo EP. **a** DMAb-αPcrV, **b** DMAb-BiSPA, **c** pGX0001 empty vector backbone, and **d** naïve muscle. Muscle tissue was harvested 3 days post DMAb injection and probed with a goat anti-humanIgG Fc antibody, followed by detection with anti-goat IgG AF88 and DAPI

**Fig. 3:**
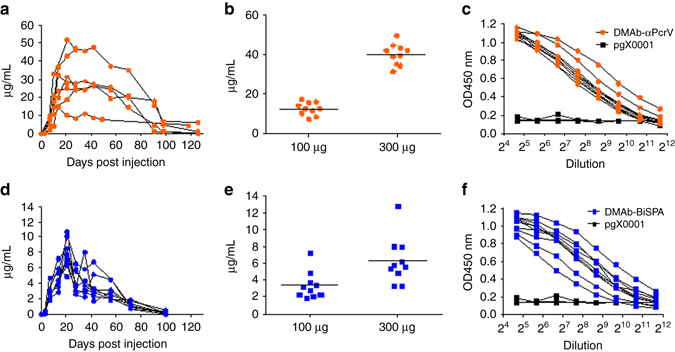
In vivo expression of DMAb-αPcrV and DMAb-BiSPA in mice. **a** B6.Cg-Foxn1 < nu > /J mice (*n* = 5/group) were administered 100 µg of DMAb-αPcrV by IM-EP. Serum levels of human IgG were monitored over 120 days. **b** Day 7 serum levels in BALB/c mice (*n* = 10/group) administered 100 and 300 µg for DMAb-αPcrV. The *line* represents the mean value. **c** Day 7 serum binding to PcrV protein in BALB/c mice (*n* = 10/group) administered 100 µg of DMAb-αPcrV. **d** B6.Cg-Foxn1 < nu > /J mice (*n* = 5/group) were administered 100 µg of DMAb-BiSPA by IM-EP. Serum levels of human IgG were monitored over 120 days. **e**, **b** Day 7 serum levels in BALB/c mice (*n* = 10/group) administered 100 and 300 µg for DMAb-BiSPA. The *line* represents the mean value. **f** Day 7 serum binding to PcrV protein in BALB/c mice (*n* = 10/group) administered 100 µg of DMAb-BiSPA

Following confirmation of in vitro expression, we investigated expression of DNA-delivered DMAb-αPcrV and DMAb-BiSPA in mice. To confirm DMAb expression in mouse muscle, anti-*P. aeruginosa* DMAb-αPcrV (100 μg), DMAb-BiSPA (100 μg), control DMAb-DVSF3 (100 μg), or control pGX0001 empty vector (100 μg) were administered to BALB/c mice by intramuscular (IM) injection in the tibialis anterior (TA), followed by intramuscular electroporation (IM-EP). Muscle tissue was harvested 3 days post injection and sections were probed with a goat anti-human IgG Fc antibody followed by detection with a donkey anti-goat IgG conjugated to AF488 (Fig. 2). Following confirmation of expression in vivo, we performed further experiments to assay DMAb levels in systemic circulation. Human IgG1 induces an anti-antibody response in immunocompetent mice, since it is recognized as non-self by the murine immune system. Therefore, we first evaluated expression in immunocompromised B6.Cg-Foxn1^nu^/J athymic mice (nude) that lack T cells and have non-functional B cells. Anti-*P. aeruginosa* DMAb-αPcrV (100 μg) or DMAb-BiSPA (100 μg) was administered to nude mice (*n* = 5/group) IM in the TA or quadriceps (quad) muscles, followed by IM-EP. Serum was collected to monitor long-term human IgG1 expression in circulation. Expression of both DMAbs was observed for 100–120 days post administration, supporting the hypothesis that these novel DNA-delivered mAbs can be produced in skeletal muscle in substantial amounts detectable in systemic circulation, with expression for several weeks (Fig. 3).

We next evaluated DMAb expression in immunocompetent BALB/c mice as they are commonly used as a model for *P. aeruginosa* infection. Mice (*n* = 10/group) were administered 100 and 300 μg doses (three injection sites × 100 µg) of DMAb-αPcrV or DMAb-BiSPA by IM-EP. The following peak DMAb expression levels were observed at day 7 following injection for DMAb-αPcrV and DMAb-BiSPA, respectively: 7.1–17.1 and 2.9–7.2 μg ml^−1^ (at the 100 μg dose), and 31.2–49.7 and 3.2–12.7 μg ml^−1^ (at the 300 μg dose) (Fig. 3b, e). Human IgG1 DMAb expression in BALB/c mice was eliminated by the mouse immune system by day 14 (Supplementary Fig. 1a, b). For comparison, a mouse IgG2a DMAb was also designed. This demonstrated long-term expression of >100 days in immune-competent BALB/c mice, without elimination by the immune system (Supplementary Fig. 1c). To confirm the target antigen specificity of DMAbs, the day 7 post administration serum was also assayed and confirmed for binding to recombinant PcrV protein by ELISA (Fig. 3c, f).

### Evaluation of DMAb-αPcrV and DMAb-BiSPA in a pneumonia model

**Fig. 4:**
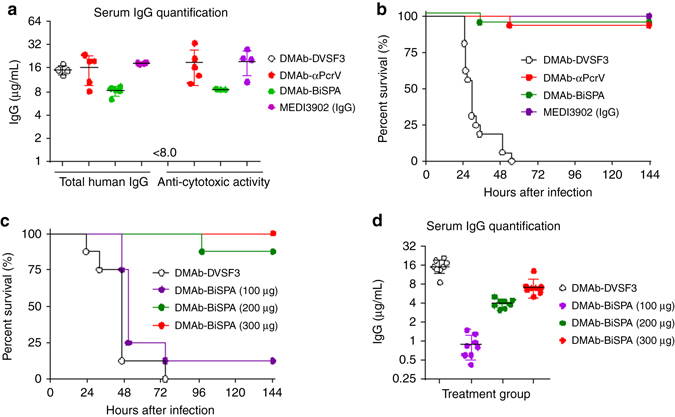
Functionality and protection by DMAb-αPcrV and DMAb-BiSPA in mice. **a** BALB/c mice were administered 300 µg of DMAb-αPcrV, DMAb-BiSPA, or MEDI3902 IgG (2 mg kg^−1^). *n* = 5 mice/group. Two animals from the DMAb-BiSPA were below the limit of detection of the anti-cytotoxic activity assay. Antibody levels are representative of DMAb in serum on the day of challenge. **b** In vivo protection in BALB/c mice following administration of control DMAb-DVSF3 (*black open circles*), DMAb-αPcrV (*red circle*), DMAb-BiSPA (*green circle*) or on day 5 or purified MEDI3902 mAb (*purple circle*) on day 1 before lethal challenge (data represented is from two independent experiments, *n* = 8/group/experiment, total *n* = 16). **c** Protection with different doses of DMAb-BiSPA: 100 µg (*purple circle*), 200 µg (*green circle*), 300 µg (*red circle*), or DMAb-DVSF3 (control). *n* = 8 mice/group. **d** Serum DMAb concentrations with different doses of DMAb-BiSPA. *n* = 8 mice/group

The in vitro and in vivo expression studies indicated that DMAbs form full, human IgG1 antibodies that bind to recombinant PcrV protein. To address the functional activity of in vivo DNA-delivered DMAbs, we evaluated protection against the highly pathogenic and cytotoxic *P. aeruginosa* strain 6077 (PA 6077), using a lethal mouse pneumonia infection model. Mice were injected 5 days before PA 6077 challenge with DMAb-αPcrV (300 μg), DMAb-BiSPA (300 μg), or an unrelated control DMAb-DVSF3 (300 μg) that targets dengue virus (DENV)^20^. We also included a positive control group in which mice received protein MEDI3902 IgG (2 mg kg^−1^) 1 day before challenge. Randomly selected animals from DMAb-αPcrV-treated and DMAb-BiSPA-treated animals were euthanized to monitor DMAb expression levels in serum at the time of challenge, as well as to evaluate the potency of the expressed DMAbs. As indicated in Fig. 4a, both monospecific DMAb-αPcrV and bispecific DMAb-BiSPA exhibited median titers of approximately 16 and 8 μg ml^−1^, respectively, when quantifying total human IgG from serum. We further evaluated the potency of in vivo expressed DMAb-αPcrV and DMAb-BiSPA by quantifying antibody expression based on the anti-cytotoxic activity from serum. No difference was observed in the quantification methods, indicating that in vivo expressed monospecific and bispecific DMAb-IgGs are fully functional and equivalent in activity in comparison to bioprocessed IgG (Fig. 4a). The remaining animals in each group were then challenged with a lethal dose of *P. aeruginosa* by intranasal inoculation followed by monitoring of survival for 6 days post infection (144 h). Animals receiving the control DMAb-DVSF3 succumbed to infection within 24–55 h. In contrast, approximately 94% of animals (15/16) that received either DMAb-αPcrV or DMAb-BiSPA survived challenge (Fig. 4b, Log-rank test *p* < 0.0001 in comparison with DMAb-DVSF3). As expected, all the positive control animals receiving MEDI3902 IgG (2 mg kg^−1^) survived challenge. In addition, treatment of mice with DMAb-BiSPA at 100 μg (1 site × 100 µg), 200 μg (2 sites × 100 µg), or 300 µg (3 sites × 100 µg) followed by infection with *P. aeruginosa*, yielded concentration-dependent survival (Fig. 4c). These results were consistent with the quantification of expressed DMAb-BiSPA in serum from these animals, in which the serum protein concentration of DMAb-BiSPA decreased with decreasing amounts of electroporated DNA (Fig. 4d).

**Fig. 5:**
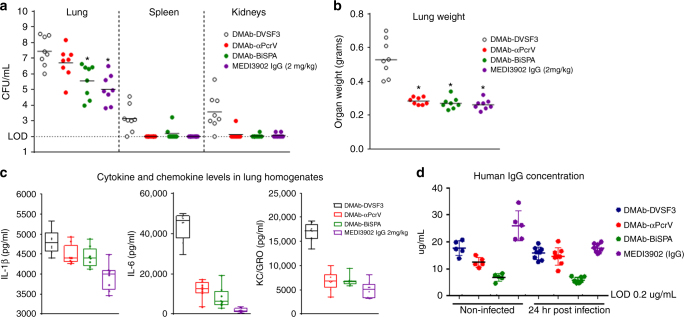
Organ protective effect of DMAb-αPcrV and DMAb-BiSPA. **a** Organ burden of *P. aeruginosa* bacteria (CFU per ml) was quantified from lung, spleen, and kidneys following lethal pneumonia challenge in animals treated with DMAb-DVSF3, DMAb-αPcrV, DMAb-MEDI3902, or MEDI3902 IgG. **b** Lung weight in infected animals following DMAb-treatment. **c** Levels of pro-inflammatory cytokines and chemokines in lung homogenates of DMAb-treated animals following lethal challenge. **a**–**c** *n* = 8 mice/group. The *line* represents the mean value. *Box* and *whisker* plots display all points and *bars* indicate minimum to maximum values. **d** Serum IgG levels of DMAb and MEDI3902 IgG in uninfected animals compared with infected animals at 24 h following lethal pneumonia challenge

We next sought to analyze the ability of anti-*Pseudomonas* DMAbs to reduce the bacterial burden in the lungs and to prevent systemic bacterial dissemination. Lungs, spleen, and kidneys were assayed 24 h post challenge with *P. aeruginosa* followed by quantification of colony forming units (CFUs) in each tissue. A significant reduction (Kruskal–Wallis with Dunn’s multiple comparison test) in CFU lung burden was observed with DMAb-BiSPA but not DMAb-αPcrV-treated animals (Fig. 5a). Importantly, bacterial burden in the lungs of DMAb-BiSPA-treated animals were similar to the lung burden observed from mice treated with protein MEDI3902 IgG and both anti-*Pseudomonas* DMAbs reduced dissemination of bacteria to the spleen and kidneys when compared to the control DMAb-DVSF3 (Fig. 5a). In addition, DMAb-αPcrV, DMAb-BiSPA, and MEDI3902 IgG were effective in preventing pulmonary edema in infected animals, as measured by lung weight, compared to control DMAb-DVSF3-treated mice (Fig. 5b). Consistent with these results, proinflammatory cytokines IL-1β and IL-6 as well as the chemokine KC/GRO (Fig. 5c) were also reduced in anti-*Pseudomonas* DMAb-treated and protein IgG-treated mice vs. the control DMAb-DVSF3. Serum IgG levels were compared between uninfected animals and infected animals 24 h post PA 6077 challenge (Fig. 5d). Taken together, these data suggest that DNA-delivered mAbs produced in vivo in skeletal muscle mediate protective activity and exhibit similar potency to exogenously produced IgG mAbs.

### Lung histopathology following challenge

**Fig. 6:**
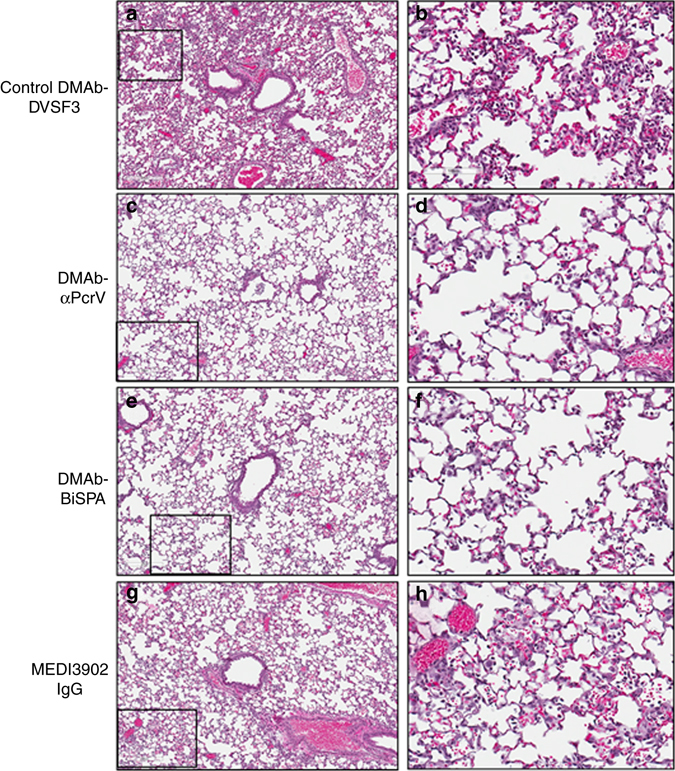
Histology of acute pneumonia at 48 h post infection with *P. aeruginosa*. **a** Post-EP with DMAb-DVSF3 showing coalescing areas of marked alveolar infiltrate and hemorrhage (10 × magnification). **b** Alveoli have marked neutrophilic infiltrates, hemorrhage, and areas of necrosis (*inset*). **c** Mild pneumonia and occasional bronchiolar debris with DMAb-αPcrV (10 × magnification). **d** Alveolar infiltrates comprises mixed neutrophilic and macrophage populations (*inset*). **e** DMAb-BiSPA group with mild alveolitis (10 × magnification). **f** Primarily neutrophilic infiltrates and mild hemorrhage in alveolar spaces (*inset*). **g** MEDI3902 IgG control demonstrates moderate alveolitis (10 × magnification). **h** Alveolar spaces contain neutrophils admixed with cellular debris and hemorrhage (*inset*). Representative data from five mice/group

Histopathology of lungs harvested at 48 h post infection demonstrated a marked alveolitis in DMAb-DVSF3-treated animals with infiltrates of neutrophils and macrophages within alveolar and perivascular spaces, along with areas of hemorrhage and alveolar necrosis. In contrast, and consistent with the reduction in proinflammatory cytokines and chemokines from lung supernatants described above, there was a clear reduction in inflammation with mild populations of primarily neutrophils and fewer macrophages in DMAb-BiSPA-treated animals with similar changes seen as well as in the DMAb-αPcrV and control MEDI3902 IgG groups (Fig. 6).

### DMAb combination with antibiotics

**Fig. 7:**
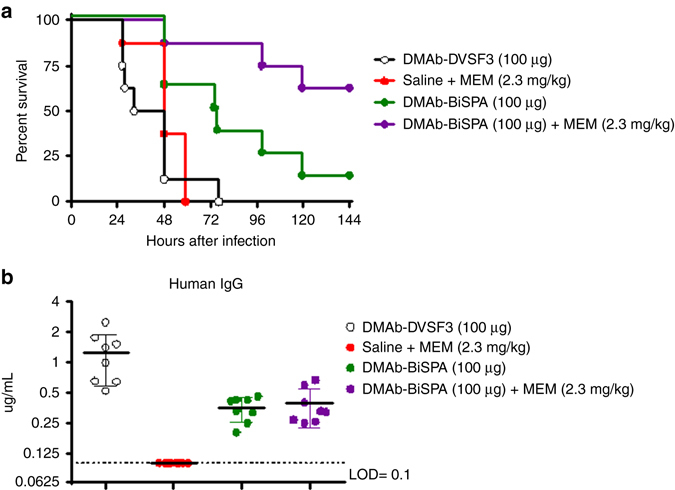
DMAb combination with antibiotic regimen. **a** BALB/c mice were injected with control DMAb-DVSF3 (100 µg), saline + MEM (2.3 mg kg^−1^), DMAb-BiSPA (100 µg), or DMAb-BiSPA (100 µg) + MEM (2.3 mg kg^−1^) and then challenged with a lethal dose of *P. aeruginosa* 6077. MEM was administered 1 h post lethal challenge. Animals were monitored for 144 h post infection. *n* = 8 mice/group. **b** DMAb serum levels in animals before lethal challenge. *n* = 8 mice/group. The *line* represents the mean value and *error bars* represent standard deviation

Broad-spectrum carbapenem family antibiotics such as meropenem (MEM) are administered when a Gram negative or *P. aeruginosa* infection is suspected. Further last-resort antibiotic regimens, such as colistin, are associated with high toxicity in humans^21, 22^ and there is the potential for the bacterium to acquire further anti-microbial resistance^23–25^. Therefore, alternative and adjunctive strategies to reduce these risks would be highly advantageous. We therefore evaluated the potential application of DMAb-BiSPA treatment in combination with MEM. For these experiments, we utilized a subtherapeutic dose of MEM (2.3 mg kg^−1^) to simulate the inadequate drug exposure encountered in patients infected with a resistant bacterium, and a subtherapeutic dose of DMAb-BiSPA (100 µg, identified in Fig. 4c). Combining these subtherapeutic dosages resulted in 67% survival compared with 10% in animals that received DMAb-BiSPA alone (Log rank test *p* = 0.026, Fig. 7). Control mice that received MEM alone or the DMAb-DVSF3 did not survive lethal challenge. Taken together, our results support the potential application of DMAb treatment as either a standalone treatment or in combination with existing antibiotic regimens. Furthermore, these data support the hypothesis that DMAb administration functions similarly to purified IgG mAbs and can mediate enhanced protective activity when combined with standard of care antibiotic treatment regimens.

## Discussion

The global health and economic impact of antimicrobial resistant infections is staggering and there is a compelling need to investigate novel strategies to prevent or treat these infections. Over 2 million cases of community-acquired and hospital-acquired resistant infections are reported each year in the United States^2^. A large percentage of these infections are caused by ESKAPE pathogens and broad-spectrum antibiotics are frequently administered as last-resort treatment^26^. However, antibiotic usage is often associated with negative side effects, including disruption of the beneficial microflora and potential for the spread of cross-resistance in non-targeted bacteria. Furthermore, inappropriate usage of antibiotics in humans or for agricultural purposes is likely a contributing factor to global resistance rates^27^. This growing predicament and the lack of novel mechanism antibiotics in the pharmaceutical pipeline has fostered the development of alternative antimicrobial strategies such as pathogen-specific mAbs. We previously reported on the derivation of a monospecific anti-PcrV mAb (V2L2MD) and bispecific anti-PcrV/Psl mAb MEDI3902 that confer potent protective activity against one of the ESKAPE pathogens, *P. aeruginosa* ^16, 17^. Using these clones as model molecules, we encoded the IgG heavy and light chain sequences into a synthetic DNA vector to test the feasibility of plasmid DNA delivery (DMAb) as an alternative mode of direct delivery of mAb in vivo to overcome some of the limitations of traditional mAb manufacturing and delivery impeding broader use.

In the current study, we demonstrated that mAbs against *P. aeruginosa* can be encoded in synthetic DNA vectors, DMAbs, and produced in vivo by skeletal muscle. The anti-*Pseudomonas* DMAbs bound effectively to therapeutic targets and were protective in a mouse model of lethal pneumonia caused by an aggressive *P. aeruginosa* strain. We identified that a single dose of DMAb is transiently expressed for 3–4 months and protection against lethal infection is comparable to treatment of mice with purified IgG. This result supports the possibility of long-term mAb administration, as DMAbs can be continuously expressed from muscle until the plasmid is eventually lost. In addition to routine administration, another foreseeable advantage for anti-*P. aeruginosa* DMAbs would be for high-risk patients with recurring infections related to chronic illnesses or implanted devices, where DMAbs may reduce the need for extended antibiotic regimens. Furthermore, we demonstrated that DMAbs can also function synergistically with a commonly used antibiotic, MEM. The synergistic effect of DMAb and antibiotic combination suggests that this strategy could have potential in reducing antibiotic treatment regimens, thereby reducing the length of antibiotic exposure in patients. This adjunctive activity is equivalent to that observed with protein IgG in previous studies^18^. Although we did not evaluate DMAb activity against a panel of *P. aeruginosa* bacterial strains, the experiments outlined in this manuscript demonstrate that DMAb is functionally equivalent to protein IgG. All bioprocessed anti-Pseudomonal IgG mAbs (anti-Psl, anti-PcrV, and MEDI3902) have been previously shown to be protective against *P. aeruginosa* clinical isolates derived from diverse serotypes, multiple T3S phenotypes (cytotoxic vs. invasive strain; ExoU^+^, ExoS^−^; ExoU^−^, ExoS^+^, respectively), and multiple infection sites^16–18, 28, 29^.

The field of mAb engineering is evolving dynamically and DMAb delivery offers an additional strategy to help transport biologically functional mAbs rapidly in vivo. In addition to obvious clinical benefits, in vivo expression of non-traditional bispecific mAb isoforms, as presented here, emphasizes the versatility of muscle to be engaged as protein production factories. Importantly, DMAb expression is transient, with similar efficacy to other therapeutic deliveries. It may be possible to develop an inducible system that will eliminate the DNA plasmid when it is no longer needed. Alternatively, DMAb DNA can potentially be re-administered indefinitely as there are no associated anti-vector responses, allowing for long-term therapy through repeat administration^30–32^. We believe that DMAb delivery represents a significant advancement not only for mAb therapy and DNA-delivery technology, but also for novel pathogen-specific treatment approaches to enhance host immunity.

Recently delivery of DNA-encoded antibodies that target Her2 in a mouse model of human breast cancer carcinoma^33^ has been reported. This study demonstrated anti-tumor efficacy comparable to protein IgG, further supporting the concept that a gene-encoded mAb can have functionality. To our knowledge, our study is the first demonstration of DNA-encoded mAb delivery that is protective against a bacterial target and the first delivery of an engineered IgG isoform. Early studies with DNA plasmid-encoded antibodies demonstrated feasibility but exhibited low-IgG expression in serum^34, 35^. Our group previously demonstrated the protective efficacy of DMAbs targeting viral infections, showing rapid protection against chikungunya^36^ and DENV infections^20^. The DMAb-targeting DENV did not promote antibody-dependent enhancement of disease. These two infectious disease models did not require high serum IgG levels; however, optimized DMAb formulations to increase expression levels are desirable so as to provide extended coverage after a single DMAb administration. Toward this end, we sought to optimize serum DMAb expression levels for our efforts against *P. aeruginosa* (Supplementary Fig. 2). This work included the inclusion of hyaluronidase into the formulation regimen, which allowed for greater IgG expression from the treated muscle.

Although further study is required for translation to humans, DMAbs are a step toward enabling routine delivery of mAb, with the potential for increasing accessibility to diverse communities worldwide. Dose translation in larger animals and humans will be important to address in future studies, particularly understanding DNA dose limitations during DMAb administration. This includes investigating different delivery and formulation optimizations that will enhance DNA expression in vivo. One strategy may be to employ other extracellular matrix enzymes to facilitate DNA entry into muscle cells^37^. Further study in non-human primates may help to understand the threshold for DNA dosage and impact on pharmacokinetic levels. Additional studies evaluating the glycosylation patterns of human IgG DMAbs produced in muscle would be beneficial to compare with bioprocessed protein IgG; however, in the context of the current study there was no difference in functionality between DMAb and its protein IgG counterpart.

In conclusion, our work described herein could be of significance for the treatment of AMR infections, particularly against ESKAPE pathogens that are refractory to many broad-spectrum antibiotic regimens. The DMAb strategy is versatile and can deliver monospecific IgGs against multiple antigenic targets as well as novel bispecific IgGs. The sustained serum mAb trough levels produced by a single dose of DMAb are consistent with functionality and protective levels afforded by bioprocessed protein IgG in vivo. The rapid development of this platform and prolonged transient expression from muscle are favorable in comparison with protein IgG mAb regimens as it could enable less frequent mAb administration. Furthermore, DMAb-encoding DNA is temperature stable allowing for transport, long-term storage, and administration to broader populations. These attractive features combined with the safety profile of DNA delivery in humans, support further DMAb studies in larger animal models as a pathogen-specific approach to targeting infectious diseases and other potential therapeutic targets.

## Methods

### Cell lines and bacteria

Human embryonic kidney (HEK) 293 T cells (ATCC CRL-3216) were maintained in Dulbecco’s Modified Eagle’s Medium (Gibco, Life Technologies, Carlsbad, CA, USA), supplemented with 10% fetal bovine serum (FBS, Atlas Biologicals, Fort Collins, CO, USA), in mycoplasma-free conditions. Routine testing was performed at the University of Pennsylvania. All cells were maintained at a low passage number. *P. aeruginosa* keratitis clinical isolate 6077 (PA 6077—provided by Dr. Joanna Goldberg—Emory University)^38^ is a cytotoxic (ExoU^+^) strain that was used for all infection experiments.

### DMAb construction and expression

The sequences of the single specificity anti-PcrV IgG (clone V2L2MD) and engineered bispecific IgG (dual specificity for PcrV and Psl, clone MEDI3902) were obtained as previously described^17, 18^. The nucleotide sequence for each human IgG1 heavy and Igκ light chains were codon optimized for both mouse and human biases to enhance expression in mammalian cells^39, 40^. Sequences were also RNA optimized for improved mRNA stability. This has been shown to result in more efficient translation on the ribosome^41, 42^, leading to increase protein yield^43^. The optimized heavy and light chain genes were then inserted into the pGX0001 DNA expression vector, under the control of a human cytomegalovirus promoter and bovine growth hormone polyA.

Both genes were encoded in *cis*, separated by a furin cleavage site (RGRKRRS) and P2A peptide. The result was two plasmids: DMAb-αPcrV and DMAb-BiSPA. HEK 293T cells were transfected with DMAb DNA using GeneJammer (Agilent, Wilmington, DE) transfection reagent. Cell supernatants and cell lysates were harvested 48 h post transfection and assayed for human IgG production by ELISA and Western blot.

### Mouse muscle tissue immunofluorescence

BALB/c mice were injected with 100 μg of DMAb by IM injection in the TA muscle followed by IM-EP. Tissue was harvested 3 days post injection, fixed in 4% Neutral-buffered Formalin (BBC Biochemical, Washington State) and immersed in 30% (w/v) sucrose (Sigma, MO) in D.I. water. Tissues were then embedded into O.C.T. compound (Sakura Finetek, CA) and snap-frozen. Frozen tissue blocks were sectioned to a thickness of 18 µm. Muscle sectioned were incubated with Blocking-Buffer (0.3% (v/v) Triton-X (Sigma), 2% (v/v) donkey serum in PBS) for 30 min, covered with Parafilm. Goat anti-human IgG-Fc fragment antibody (A-80-104A, Bethyl, TX) was diluted 1:100 in incubation buffer (1% (w/v) BSA (Sigma), 2% (v/v) donkey serum, 0.3% (v/v) Triton-X (Sigma), and 0.025% (v/v) 1g ml^−1^ Sodium Azide (Sigma) in PBS). 50 µl of staining solution was added to each section and incubated for 2 h. Sections were washed 5 min in 1×PBS three times. Donkey anti-goat IgG AF488 (ab150129, Abcam, USA) was diluted 1:200 in incubation buffer and 50 µl was added to each section. Section were washed after 1 h incubation and mounted with DAPI-Fluoromount (SouthernBiotech, AL) and covered.

In vivo expression of DMAb constructs was imaged with a BX51 Fluorescent microscope (Olympus) equipped with Retiga3000 monochromatic camera (QImaging).

### Human IgG quantification by ELISA and by anti-cytotoxic activity

Ninety-six-well, high-binding immunosorbent plates were coated with 10 µg ml^−1^ purified anti-human IgG-Fc (A-80-104A, Bethyl Laboratories, Montgomery, TX) and incubated overnight at 4 °C. The following day, plates were washed and blocked at room temperature for at least 1 h with PBS containing 10% FBS. Samples were serially diluted two-fold and transferred to the blocked plate and incubated for 1 h at room temperature. Purified human IgGκ (P80-111, Bethyl Laboratories, Montgomery, TX) was used as a standard. Following incubation, samples were probed with an anti-human IgGκ antibody conjugated to horseradish peroxidase (A80-115P, Bethyl Laboratories, Montgomery, TX) at a 1:20,000 dilution. Plates were developed using o-Phenylenediamine dihydrochloride substrate (OPD, Sigma Aldrich, St. Louis, MO) and stopped with 2N H_2_SO_4_. A BioTek Synergy2 plate reader (Bioteck, Winooski, VT) was used to read the plates at OD450 nm. Alternatively, human IgG from serum was quantified as described above with the exception of using an anti-idiotype mAb (0.05 μg per well suspended in 0.2 M sodium bicarbonate buffer, pH 9.4) specific for V2L2MD or MEDI3902 as the capture reagent. Purified V2L2MD or MEDI3902 was used as a standard.

DMAb was also quantified from serum using 384-well black MaxiSorp plates (Nalge, Nunc, Thermo Fisher Scientific, Waltham, MA) coated with 10 μg ml^−1^ Goat anti-Human IgG (H + L) (31410, Pierce, Thermo Fisher). Plates were washed and blocked for 1–2 h at room temperature with Blocker Casein in PBS (Thermo Fisher). After blocking, a standard containing MEDI3902 or V2L2MD was serially diluted 1:2 across the plate, while serum samples were diluted 1:20, 1:40, 1:80, and 1:160. Plates containing the samples were then incubated for 1 h at room temperature. After washing, plates were probed with Donkey anti-Human IgG-HRP (709-035-149, JacksonImmuno Research, West Grove, PA) at a 1:4000 dilution and incubated for 1 h at room temperature. After washing, the immune reaction was developed by adding SuperSignal ELISA Pico Reagent (Thermo Fisher) and fluorescence was read on the Perkin Elmer Envision.

DMAb was also quantified from serum based on anti-cytotoxic activity mediated by DMAb-αPcrV and DMAb-BiSPA that measures the protection of A549 cells (ATCC® CCL-185™) from the cytotoxic effects of PA 6077. The activity of mouse serum was compared to a standard curve of naïve mouse serum spiked with V2L2MD IgG.

### Binding ELISA

Ninety-six-well plates immunosorbent plates were coated overnight with *Pseudomonas* PcrV protein (MedImmune, Gaithersburg, MD) at 0.5 µg ml^−1^. The following day, serum samples from DMAb-administered animals were serially diluted two-fold and then transferred to the blocked plate. Samples were probed with an anti-human IgG H + L antibody conjugated to HRP (SAB3701359, Sigma Aldrich, St. Louis, MO) at a dilution of 1:5000 and developed with OPD substrate.

### Western blot

The cell lysates from DMAb-transfected cells were collected in cell lysis buffer (Cell Signalling Technologies, Danvers, MA). Samples were centrifuged at 16,000 × *g* and the supernatant containing the protein fraction was collected. The samples were quantified using a bicinchoninic acid assay (Pierce, Thermo Fisher Scientific) and 10 µg total lysate was loaded on a 4–12% Bis-Tris SDS-PAGE gel (NuPAGE, Thermo Fisher Scientific). The gel was transferred to a nitrocellulose membrane using the iBlot2 system (Thermo Fisher Scientific). The membrane was blocked in 5% powdered skim milk + 0.5% Tween-20 and then probed using a donkey anti-human H + L antibody conjugated to HRP (SAB3701359, Sigma Aldrich, 1:5000 dilution). Bands were developed using a chemiluminescent system (ECL, GE Healthcare, UK) and visualized on film.

### Mice

Female, 6–8-week-old B6.Cg-Foxn1nuJ and BALB/c mice were purchased from The Jackson Laboratory (Bar Harbor, ME) and housed in the animal facilities at the University of Pennsylvania or MedImmune, AstraZeneca. All animal protocols were approved by the institutional University of Pennsylvania and MedImmune Institutional Animal Care and Use Committees (IACUC), following guidelines from Association for Assessment and Accreditation of Laboratory Animal Care International. Further IACUC oversight was provided by The Animal Care and Use Review Office, required for DARPA-funded research. Animals received an IM pre-injection of hyaluronidase (400 U ml^−1^, Sigma Aldrich) 30 min–1 h before IM injection of 100–300 µg DMAb-αPcrV or DMAb-BiSPA in the TA or quad muscles, followed by electroporation (IM-EP). Serum levels of DMAbs were monitored following administration.

### Lethal pneumonia challenge

BALB/c mice (*n* = 8/group) received 100 µg or 300 µg of DMAb-αPcrV or DMAb-BiSPA by IM-EP at day 5 before challenge. The unrelated DENV DMAb-DVSF3^20^ was included as a control. A fourth group of animals received an intraperitoneal injection of purified protein IgG MEDI3902 (2 mg kg^−1^) on day −1 before challenge. On day 0, animals received an intranasal challenge of 9.75e5–1.0e6 CFUs of the aggressive, anti-microbial resistant *P. aeruginosa* strain 6077. Animals were monitored for 6 days following intranasal challenge for survival as described in ref. ^16^. Briefly, animals were anesthetized with ketamine and xylazine followed by intranasal administration of the bacterial inoculum contained in 0.05 ml. For organ burden analyses, lungs, spleens, and livers were harvested from DMAb-treated animals 24 h post infection followed by homogenizing and plating of Luria agar plates for enumeration of bacterial CFU. IL-1β, IL-6, and KC/GRO were quantified from the supernatant of lung homogenates using a multiplex kit (Meso Scale Diagnostics), according to the manufactures instructions. For DMAb and MEM combination experiments, MEM was administered subcutaneously 4 h after infection.

### Histopathology

Lungs were harvested at 48 h post infection and fixed in 10% neutral buffered formalin (VWR, Radnor, PA) for a minimum of 48 h. Fixed tissues were then routinely processed and embedded in paraffin, sectioned at 3-µm thickness, and stained with Gill’s hematoxylin (Mercedes Medical, Sarasota, FL) and eosin (Surgipath, Richmond, IL) for histologic evaluation by a pathologist blinded to the experimental conditions.

### Statistics

All statistical analyses were peformed using GraphPad Prism 6.0 software (La Jolla, CA) or SPSS (IBM). Sample size calculations for two independent proportions were calculated with alpha 0.05 and power 0.90. A minimum of *n* = 5 mice was calculated to be needed in order to ensure adequate power. We performed student’s *t*-test or one-way analysis of variance (ANOVA) calculations, where necessary. Survival data was represented by a Kaplan–Meier survival curve and significance was calculated using a log-rank test and one-way ANOVA with correction for multiple comparisons. These data were considered significant if *p* < 0.05. The lines in all graphs represent the mean value and error bars represent the standard deviation. No samples or animals were excluded from the analysis. Randomization was not performed for the animal studies. Samples and animals were not blinded before performing each experiment.

### Data availability

The nucleotide sequences for the optimized human IgG1 heavy and Igκ light chains have been deposited in the NCBI Nucleotide database with accession codes LQ055727.1, LQ055728.1, LQ055748.1, and LQ055750.1. The authors declare that all other relevant data supporting the findings of the study are available in this article and its Supplementary Information files, or from the corresponding author upon request.

## Electronic supplementary material

Supplementary Information
