# Supplementary material for: An engineered bispecific DNA-encoded IgG antibody protects against Pseudomonas aeruginosa in a pneumonia challenge model
Source: Nat Commun. 2017 Sep 21;8:637. doi: 10.1038/s41467-017-00576-7 (PMC5608701; doi:10.1038/s41467-017-00576-7)

File name: Supplementary Information
Description: Supplementary Figures

Supplementary Figure 1. Pharmacokinetics of DMAb-αPcrV, DMAb-BiSPA, and a mouse IgG2a DMAb in BALB/c mice. BALB/c mice received a 100 µg DNA injection of DMAb-αPcrV, DMAb-BiSPA, or control mouse IgG2a DMAb into the TA muscle, followed by *in vivo* electroporation (n=10/group). Serum human IgG1 levels were monitored for 21 days following DMAb injection and quantified by ELISA. Mouse IgG2a levels were monitored for 103 days following DMAb injection and quantified by ELISA.

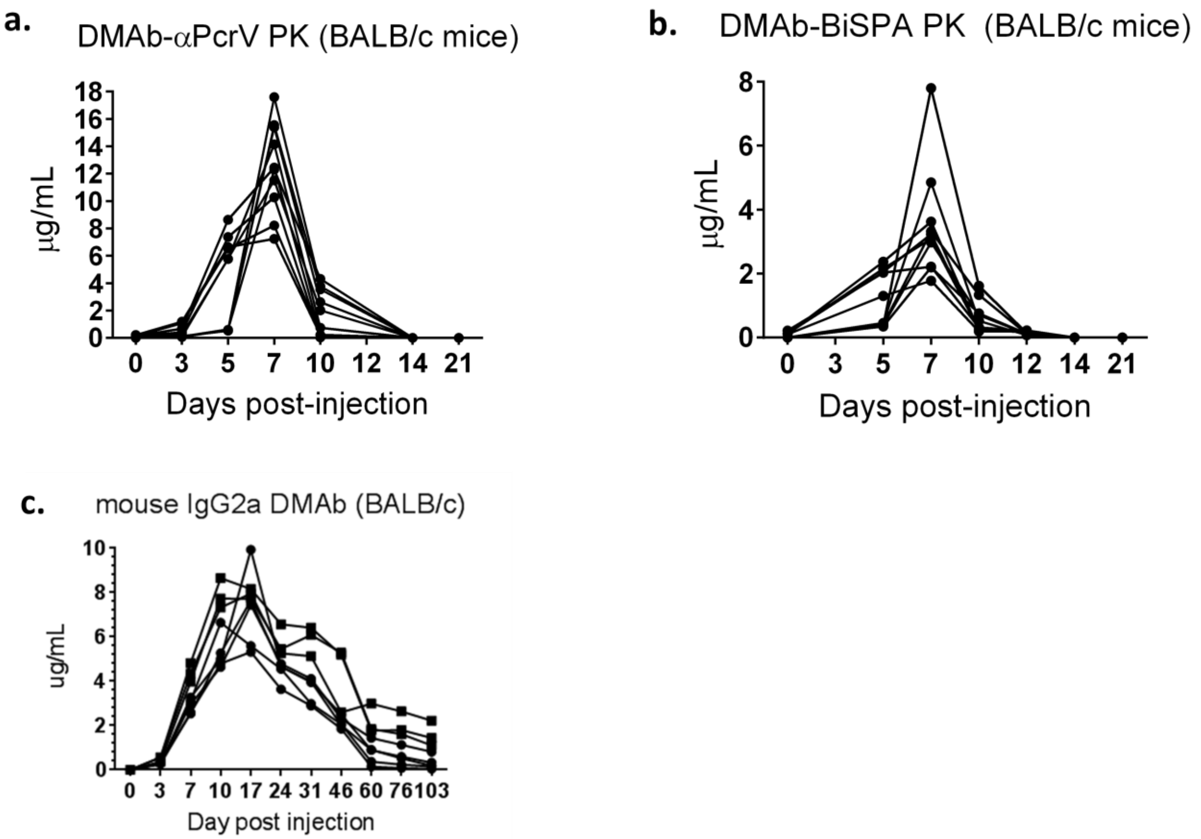

Supplementary Figure 2. Optimization of DMAb-V2L2 *in vivo* expression. BALB/c mice received a single DNA injection into the TA muscle with DMAb-αPcrV or DMAb-BiSpA DNA followed by *in vivo* electroporation. Graph represents Day 7 serum levels in BALB/c mice (n=5/group) administered 100 µg, 200 µg, or 300 µg for DMAb-αPcrV, respectively, before and after sequence, formulation with hyaluronidase (400U/mL), and electroporation optimizations. Error bars represent the standard deviation.

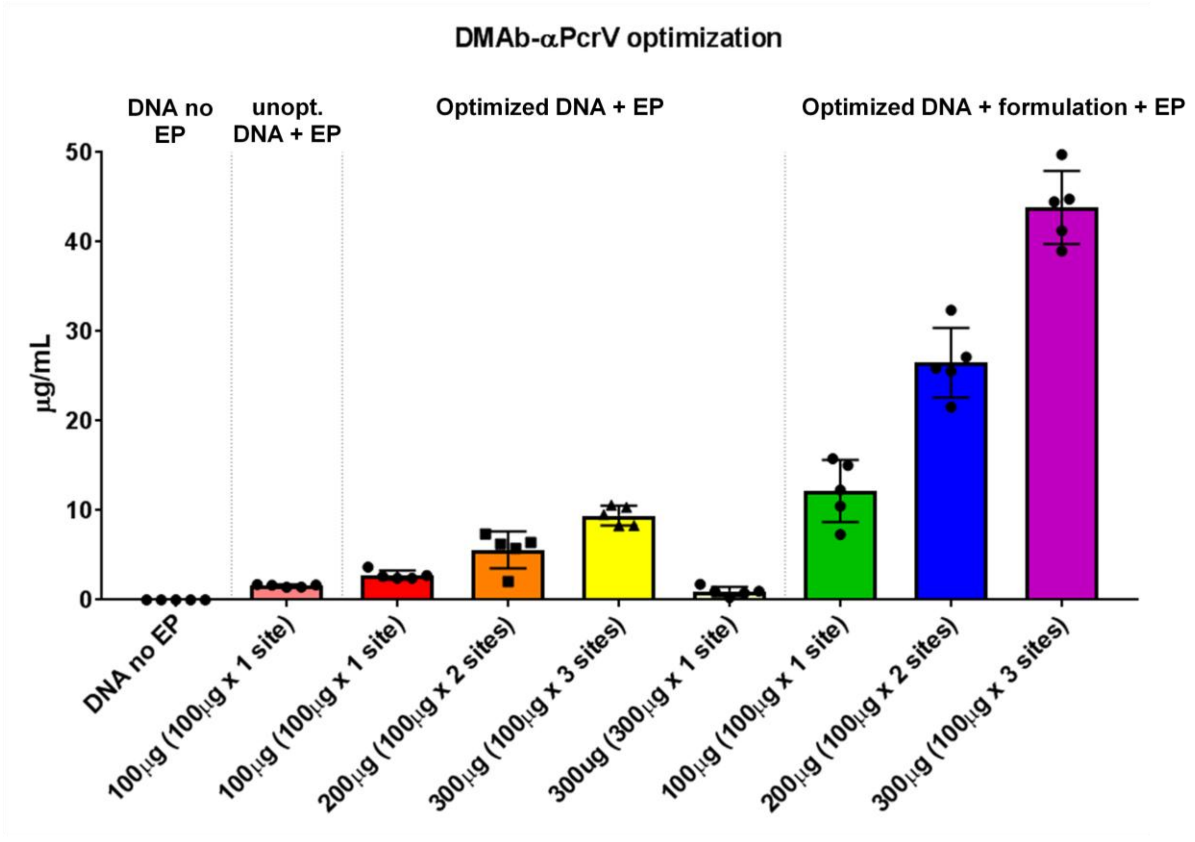

Supplement: Supplementary file 1 — Supplementary Information [file 41467_2017_576_MOESM1_ESM.pdf]
